# Syphilitic Tonsillitis: A Case Report and Review of the Literature

**DOI:** 10.7759/cureus.105613

**Published:** 2026-03-21

**Authors:** Alexandros Ganaiem, Aggeliki Grigoraki, Kallirroi Spanou, Elisavet Kanna, Spyridon Potamianos

**Affiliations:** 1 Otolaryngology - Head and Neck Surgery, Athens General Hospital of Thoracic Diseases "Sotiria", Athens, GRC; 2 Medical School, National and Kapodistrian University of Athens, Athens, GRC; 3 Anaesthesiology, "Hippokration" General Hospital of Athens, Athens, GRC; 4 Pathology, Pathlabs, Athens, GRC; 5 Pediatric Surgery, Panagiotis and Aglaia Kyriakou Childrens' Hospital, Athens, GRC; 6 Otolaryngology - Head and Neck Surgery, Evgenidio Hospital, Athens, GRC

**Keywords:** atypical secondary syphilis, early syphilis, histopathology of syphilis, oropharyngeal syphilis, syphilis, syphilis screening, syphilis screening algorithm, tonsilar syphilis

## Abstract

Syphilitic tonsillitis is an uncommon manifestation of Treponema pallidum infection that may present with nonspecific oropharyngeal symptoms and mimic other infectious or neoplastic processes. We report a case of a 30-year-old man who presented with a two-month history of sore throat, dysphagia, low-grade fever, and hoarseness. Clinical examination revealed bilateral tonsillar hypertrophy with ulcerated papules and cervical lymphadenopathy. A tonsillectomy was performed due to poor treatment response. Histopathology showed mucosal ulceration with architectural effacement due to marked paracortical hyperplasia, accompanied by prominent plasma cell infiltration (CD138+), scattered giant cells, and small non-caseating epithelioid granulomas. Serologic testing confirmed the diagnosis of oropharyngeal syphilis. The patient was treated with a single dose of 2.4 million units of intramuscular benzathine penicillin G, resulting in marked clinical improvement and a significant decline in nontreponemal titers at follow-up. This case underscores the diagnostic challenge of syphilitic tonsillitis and highlights the importance of considering syphilis in patients with persistent oropharyngeal lesions to avoid disease progression and complications.

## Introduction

Syphilis is a chronic infectious disease caused by Treponema pallidum, primarily transmitted through sexual contact, as well as through blood exposure and transplacental spread [1]. While its classical manifestations include genital chancres and systemic mucocutaneous lesions, the clinical presentation can be highly variable, with oropharyngeal involvement increasingly recognized in recent literature [2].

Tonsillar involvement in syphilis is uncommon and may present as ulceration, exophytic lesions, or mass-like hypertrophy, mimicking bacterial tonsillitis, malignancy, or other infectious etiologies. Some reported cases occur during the secondary stage, where systemic dissemination of the organism results in widespread mucocutaneous involvement. Primary syphilitic chancres of the tonsil are painless, and tertiary stage tonsillar gummas show chronic inflammation [2].

Given the global rise in syphilis cases and its varied symptoms, awareness of atypical presentations is critical [3]. Syphilis is classically referred to as the “Great Imitator”. In this report, we present a case of secondary syphilis initially misdiagnosed as bacterial tonsillitis, with the diagnosis indicated after tonsillectomy and histopathological evaluation, and confirmed by serologic testing and PCR detection of Treponema pallidum. This case emphasizes the importance of considering syphilis in the differential diagnosis of persistent oropharyngeal lesions, especially when standard therapy fails.

This article was previously presented as a poster at the 23rd Panhellenic Congress of Otolaryngology-Head and Neck Surgery on February 26, 2026.

## Case presentation

A 30-year-old man who has sex with men (MSM) presented to our clinic with symptoms of sore throat, dysphagia, low-grade fever, and hoarseness persisting for two months. An upper respiratory tract infection was originally diagnosed by an internist, who had prescribed antibiotic therapy consisting of amoxicillin/clavulanate for potential bacterial tonsillitis. Subsequently, the patient developed a non-pruritic maculo-papular eruption involving the trunk, palms, and soles, initially attributed to an allergic reaction to antibiotic therapy, therefore the treatment was replaced with oral clindamycin, which was administered for an additional 10 days. 

Despite treatment, the patient’s condition worsened, prompting a visit to our clinic. At presentation, the patient had a hot potato voice. On clinical examination of the head and neck, he exhibited unilateral tonsillar enlargement with ipsilateral cervical lymphadenopathy. Routine blood laboratory tests were normal as below, including 3% eosinophilia (Table 1). Serologic testing for hepatitis B and C viruses (HBV, HCV), Toxoplasma, and human immunodeficiency virus (HIV) revealed no evidence of acute or active infection. Ebstein-Barr virus (EBV) and cytomegalovirus antibody seropositivities were consistent with past exposures and old infections. Anti-HBV surface antibodies were consistent with prior vaccination (Table 2). 

**Table 1:** Laboratory test results Reference ranges may vary slightly depending on the laboratory.

Laboratory test	Result	Reference Range
Hemoglobin (Hb)	14.20 g/dL	13.50-17 g/dL
White Blood Cells (WBC)	7.60 x 10⁹ /L	4-10 x 10⁹ /L
Neutrophils (NEUT%)	58%	40-70%
Lymphocytes (LYMPH%)	32%	20-45%
Monocytes (MONO%)	6%	2-10%
Eosinophils (EO%)	3%	1-6%
Basophils (BASO%)	1%	0.5-2%
C-Reactive Protein (CRP)	1.20 mg/L	<5 mg/L
Erythrocyte Sedimentation Rate (ESR)	10 mm/h	0-20 mm/h
Aspartate Aminotransferase (AST / SGOT)	22 U/L	10-40 U/L
Alanine Aminotransferase (ALT / SGPT)	24 U/L	7-56 U/L

**Table 2:** Serological test results Reference ranges may vary slightly depending on the laboratory. EBV VCA: Epstein-Barr virus viral capsid antigen, CMV: cytomegalovirus, HCV: hepatitis C virus, Ig: immunoglobulin

Serology test	Result	Interpretation	Reference Range
Hepatitis B surface antigen (HBsAg)	0.11 S/CO	Negative	<1.0 S/CO (Negative)
Hepatitis B core antibody (Anti-HBc, total)	0.15 S/CO	Negative	<1.0 S/CO (Negative)
Hepatitis B surface antibody (Anti-HBs)	52 mIU/mL	Positive / immune	≥10 mIU/mL (protective immunity)
Anti-HCV antibody	0.07 S/CO	Negative	<1.0 S/CO (Negative)
EBV heterophile antibodies (Monospot)	Negative (qualitative)	Negative	Positive / Negative (qualitative)
EBV VCA IgM (anti-viral capsid antigen antibody)	0.18 Index	Negative	<0.90 Index (Negative)
EBV VCA IgG (anti-viral capsid antigen antibody)	3.50 Index	Positive / past infection	>1.0 Index (Positive/past)
IgG to Epstein-Barr nuclear antigen (EBNA antibody)	4.20 Index	Positive / past infection	>1.0 Index (Positive/past)
Anti-CMV IgG antibodies	2.80 Index	Positive / past infection	>1.0 Index (Positive/past)
Anti-CMV IgM antibodies	0.20 Index	Negative	<0.90 Index (Negative)
Toxoplasma gondii IgG antibodies	0.12 IU/mL	Negative	<1.0 IU/mL (Negative)
Toxoplasma gondii IgM antibodies	0.15 Index	Negative	<0.90 Index (Negative)
HIV combo I, II, p24 (HIV-1 p24 antigen and HIV-1/2 antibodies)	0.09 S/CO	Negative	<1 S/CO (Negative)

A contrast-enhanced neck CT confirmed known tonsillar hypertrophy and multiple ipsilateral level IIa lymph nodes, the largest of which was 3cm. Given that lymphoma and tonsillar malignancy were included in the initial differential diagnosis, tonsillectomy under general anesthesia was scheduled to establish a definitive diagnosis.

In the 10-day interval between diagnosis and surgery, the patient’s symptoms of dysphagia and hoarseness progressed and bilateral tonsillar hypertrophy classified as Grade 4 was noted with ulcerated papules on both tonsils.

The patient finally underwent bilateral tonsillectomy for diagnostic as well as for palliative purposes. Intraoperatively pathologic tissue was identified and removed. The postoperative course was uncomplicated, and the patient mentioned that he was relieved from his symptoms.

**Figure 1:**
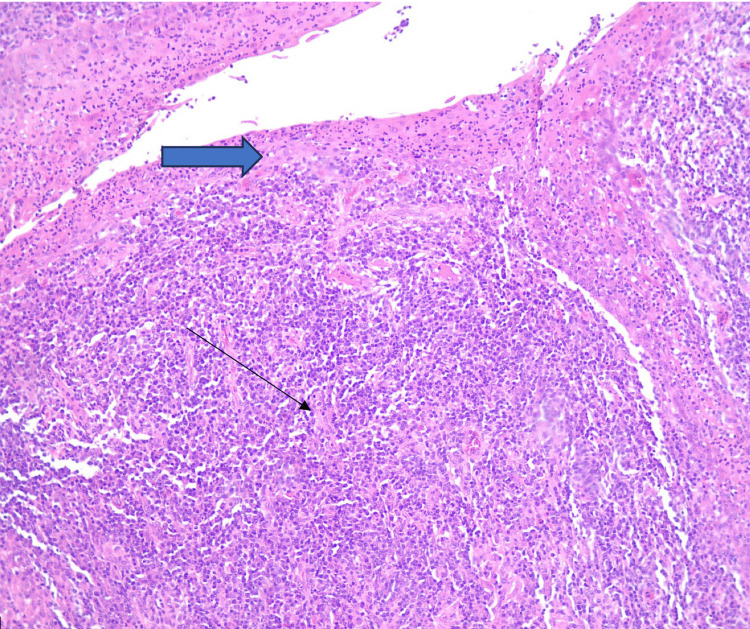
(H&E; ×10) Notice the ulceration of the mucosa (wide arrow) with effaced architecture due to accentuated paracortical hyperplasia (black arrow). (H&E; ×10): Hematoxylin and Eosin stain, ×10 magnification

**Figure 2:**
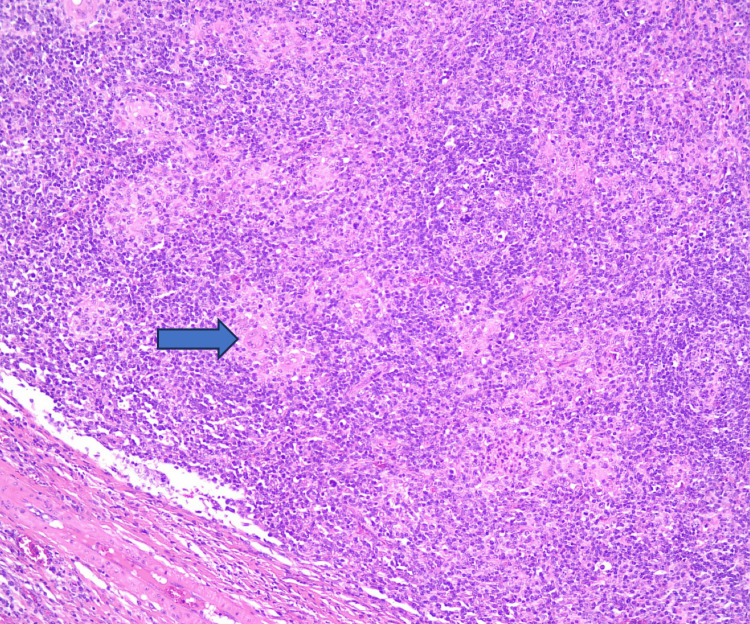
(H&E; ×10) There is an outstanding infiltration of plasma cells, isolated giant cells and small non-caseating epithelioid granulomas (wide arrow). (H&E; ×10): Hematoxylin and Eosin stain, ×10 magnification

**Figure 3:**
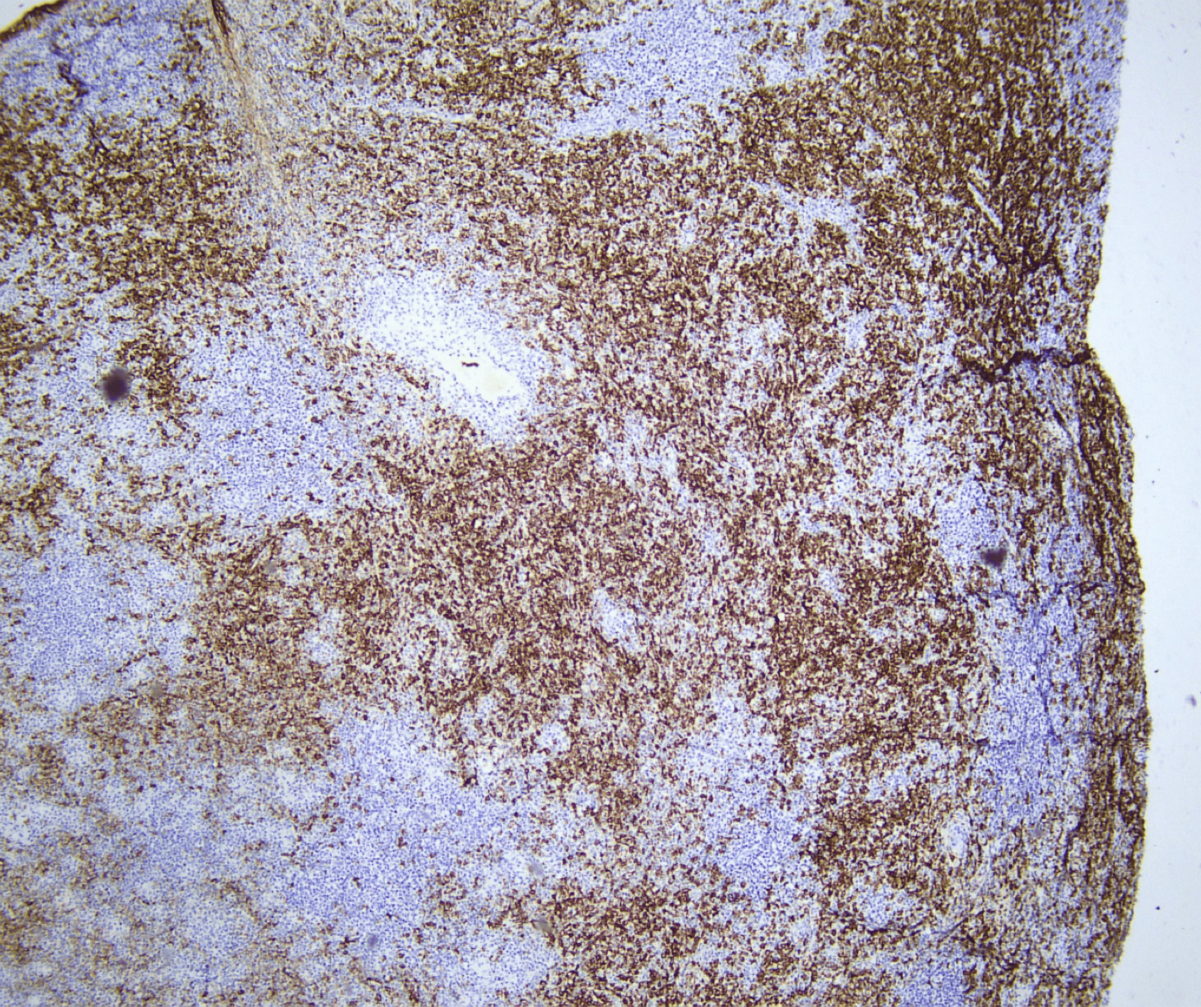
CD138 Immunohistochemistry ×4 magnification. Οutstanding abundance of plasma cell infiltration.

The histopathological examination revealed mucosal ulceration with effacement of the normal architecture due to marked paracortical hyperplasia. Prominent plasma cell infiltration, scattered giant cells, and small non-caseating epithelioid granulomas were observed (Figures 1, 2). In addition, CD138 immunohistochemistry highlights abundant plasma cell infiltration (Figure 3). Given these findings and the suspicion of syphilitic infection, the specimen was submitted for further molecular analysis by PCR, which confirmed the presence of Treponema pallidum DNA.

Subsequently, serology testing was performed, demonstrating a positive Treponema pallidum particle agglutination (TPPA) test with reactive titers 1:320 and a positive non-treponemal venereal disease research laboratory (VDRL) test with reactive titers 1:16, further supporting the diagnosis of early active syphilis (Table 3).

**Table 3:** Serological Tests for Syphilis: Treponemal and Non-treponemal Assays Reference ranges may vary slightly depending on the laboratory. TPPA: Treponema pallidum particle agglutination, VDRL: venereal disease research laboratory

Category	Serology test	Titers	Result - Interpretation	Reference Range
Treponemal test	TPPA	1:320	Reactive - Positive	Titers >1:40 (reactive)
Non-treponemal test	VDRL	1:16	Reactive - Positive	Titers ≥1:1 (reactive)

The man was treated with benzathine penicillin (2.4 million units intramuscular) and asked to return for review and serological testing to assess for treatment response after four weeks. He was also asked to abstain from sex for two weeks, and two weeks after his partner was tested and treated for syphilis. At three-month follow-up, the patient was asymptomatic, with non-treponemal serological test turning negative at six months with VDRL non-reactive (titers <1:1).

## Discussion

Syphilis is a sexually transmitted infection caused by Treponema pallidum, transmitted mainly via direct contact with syphilitic lesions, but also via blood exposure and transplacental passage. Its incidence declined with the introduction of penicillin in the middle of the 20th century but has resurged since the 1990s, particularly among HIV-positive MSM [1]. The World Health Organization (WHO) estimated 19.9 million prevalent cases and 6.3 million new cases of syphilis among individuals aged 15-49 years in 2016. As of 2014, median incidence rates were 17.7 per 100,000 males and 17.2 per 100,000 females [3]. 

The infection progresses through defined stages. Primary syphilis appears nine to 90 days after exposure (commonly three weeks) as a painless chancre, sometimes accompanied by regional lymphadenopathy. Atypical presentations may involve multiple or painful lesions and the most frequent site of involvement is the genital area, followed by the oral cavity. Lesions usually resolve within three to six weeks, even without treatment [2]. Secondary syphilis develops four to 10 weeks after the primary lesion in approximately 25% of untreated individuals, presenting with systemic symptoms such as maculopapular rash (often involving the palms and soles), mucocutaneous lesions, generalized lymphadenopathy, condyloma lata, patchy alopecia, fever, and occasionally arthralgias, nephritis, or hepatitis. The manifestations often resolve spontaneously, except in severe forms such as lues maligna, which presents with necrotic ulcerative skin lesions. Latent syphilis is asymptomatic but detectable serologically and is classified as early (less than one year) or late (more than one year). Tertiary syphilis may arise years later (one to 30 years), causing gummatous lesions, cardiovascular complications, and neurologic dysfunction. Like oropharyngeal syphilis, neurologic, ocular, and otic manifestations can arise at any stage [4-7].

Head and neck mucosal involvement most often affects the oral cavity and oropharynx [8]. The tonsils represent the most common oropharyngeal site, accounting for approximately 71% of cases (71% unilateral, 29% bilateral), followed by the lateral and posterior pharyngeal wall. Tonsillar involvement may arise through direct inoculation, such as oral-genital contact, or as part of secondary mucous patches [5]. Primary and tertiary syphilis can present rarely with a solitary chancre or gummatous lesions of the tonsil [9-12].

The diagnosis of syphilitic tonsillitis can be particularly challenging due to its clinical similarity to common causes of tonsillitis and malignant oropharyngeal diseases. Symptoms such as sore throat, odynophagia, dysphagia, cervical lymphadenopathy, fever, and malaise are nonspecific and often attributed to bacteria like Streptococcus pyogenes or viral etiologies such as EBV and adenovirus. In addition, syphilitic tonsillitis may also present as unilateral tonsillar enlargement, with exudate, ulcerative, exophytic, or mass-like lesions, that may be painless and mimic neoplasms. Misattribution of cutaneous rashes or failure to recognize Jarisch-Herxheimer inflammatory reactions to beta-lactam antibiotics as drug reactions, as in our patient, can further delay recognition. These overlaps highlight the need for clinicians to maintain a high index of suspicion, especially in cases unresponsive to standard antibiotic therapy. A careful patient history and persistent clinical monitoring is needed to consider syphilis in the differential diagnosis of refractory or atypical tonsillar disease.

Since Treponema pallidum cannot be cultured routinely, serologic testing remains the gold standard for screening, diagnosis, and monitoring. Nontreponemal tests, such as VDRL, rapid plasma reagin (RPR) and toluidine red unheated serum (TRUST), are quantitative tests detecting antibodies against cardiolipin and other lipoidal antigens. They are less specific but low-cost and useful for initial screening. Treponemal tests, like TPPA, T. pallidum enzyme immunoassay (TP-EIA), chemiluminescence immunoassay (CIA), and fluorescent treponemal antibody absorbed (FTA-ABS), are qualitative tests, confirming antibodies specific to T. pallidum [13]. Both are required for diagnosis, as false positives and negatives can occur, particularly in early or late disease and immunocompromised patients [14]. 

The antibody titers of non-treponemal tests are usually used to assess disease activity, monitor response to treatment, and guide follow-up. Nontreponemal titers, especially VDRL, are typically reactive during the primary stage of the infection, and increase further in the secondary and early latent stage, reaching values between 1:8 and 1:32 or higher. Titers typically peak after three weeks and may subsequently plateau or decline in later months or years, depending upon host immunity and antibiotic exposures. A VDRL titer of 1:16 is compatible with active infection in early syphilis and is higher than titers observed in false-positive reactions, which are usually ≤1:4. In contrast, treponemal tests indicate prior or current exposure to the pathogen, but do not always reflect disease activity. Treponemal tests are primarily used as a specific confirmatory test following a positive screening result. Although a fourfold decrease in treponemal titers (TPPA) after treatment is generally considered indicative of adequate treatment response, these tests are not typically used for monitoring, unless the patient suffers reinfection. Barring reinfection, treponemal antibody titers decline over decades but remain detectable indefinitely if not for life [13,14].

In our patient, the diagnosis was established histologically following tonsillectomy. Direct detection of T. pallidum from tissue biopsy - via darkfield microscopy, direct fluorescent antibody testing for T pallidum (DFA-TP), PCR, or Warthin-Starry and Dieterle staining - can provide definitive diagnosis, especially in atypical oropharyngeal manifestations. PCR is highly sensitive, and IgM immunoblotting may be useful when lesions are absent. Histopathological findings are often nonspecific and may include a plasma cell-rich infiltrate, multinucleated giant cells, non-caseating epithelioid granulomas, or a lymphohistiocytic infiltrate [10,15]. In the present case, silver staining techniques were not performed, as PCR-based detection was available and considered sufficient for definitive identification. The diagnosis was further supported by serologic testing performed following histopathological evaluation. The positive results (VDRL titer 1:16 and TPPA titer 1:320) were consistent with active syphilis. 

Benzathine penicillin G remains the first-line therapy for all stages of syphilis. Primary, secondary, or early syphilis is treated with one to three doses of IM benzathine penicillin G, each dose 2.4 million U, with follow-up in order to avoid complications including neurosyphilis. Late latent or tertiary disease requires 2.4 million units IM weekly for three weeks. Neurologic, ocular, or otic involvement requires intravenous penicillin G for 10 to 28 days. Alternatives for non-pregnant, penicillin-allergic patients include doxycycline or ceftriaxone, though desensitization or rechallenge is preferred in severe high-risk cases or treatment failure. Jarisch-Herxheimer reactions should be managed symptomatically [7]. Follow-up consists of clinical assessment and nontreponemal serologic testing at six, 12, and 24 months, with a nonreactive result or a ≥4-fold titer decline indicating adequate treatment response. Of course, HIV testing is recommended for all patients. Reported cure rates for early syphilis approach 90-100%, regardless of HIV status [7,15].

Clinical awareness and prompt recognition are crucial to prevent misdiagnosis and ensure timely treatment to avoid disease progression and complications.

## Conclusions

Syphilitic tonsillitis is a clinically significant manifestation of syphilis that may closely mimic other infectious or malignant conditions. This case underscores the importance of maintaining a broad differential diagnosis when evaluating persistent oropharyngeal symptoms, especially in patients with systemic features or treatment failure. Increased awareness among clinicians is essential to prevent misdiagnosis because untreated infection can lead to irreversible complications.
